# Changes in adverse pregnancy outcomes in women with advanced maternal age (AMA) after the enactment of China’s universal two-child policy

**DOI:** 10.1038/s41598-022-08396-6

**Published:** 2022-03-23

**Authors:** Hui Li, Nawsherwan, Cuifang Fan, Shanshan Yin, Ijaz ul Haq, Sumaira Mubarik, Ghulam Nabi, Suliman Khan, Linlin Hua

**Affiliations:** 1 Advanced Medical Center, The Second Affiliated Hospital of Zhengzhou University, Zhengzhou, Henan, China; 2 Department of Medicine, Taixing People Hospital, Taizhou, Jiangsu China; 3 Department of Preventive Medicine, School of Health Sciences, Wuhan University, Wuhan, Hubei China; 4 Department of Obstetrics and Gynecology, Renmin Hospital, Wuhan University, Wuhan, Hubei China; 5 Henan Academy of Medical Sciences, Zhengzhou, Henan China; 6 Department of Public Health and Nutrition, The University of Haripur, Haripur, Pakistan; 7 Department of Epidemiology and Biostatistics, School of Health Sciences, Wuhan University, Wuhan, Hubei China; 8 Institute of Nature Conservation, Polish Academy of Sciences, Krakow, Poland

**Keywords:** Health care, Medical research

## Abstract

The universal two-child policy (TCP; 2016) in China has affected many aspects of maternal-neonatal health. A tertiary hospital-based retrospective study (2011–2019) was used to find the association of these policy changes with maternal age and pregnancy outcomes in women with AMA (≥ 35 years) in the Hubei Province, China. The proportion of neonatal births to women with AMA increased by 68.8% from 12.5% in the one-child policy (OCP) period to 21.1% in the universal TCP period [aOR 1.76 (95% CI: 1.60, 1.93)]. In the univariate analysis, the proportion of preterm births (29.4% to 24.1%), low birth weight (LBW) (20.9% to 15.9%), and hypertensive disorders of pregnancy (HDP) (11.5% to 9.2%) significantly (*p* < 0.05) decreased in women with AMA from the OCP period to universal TCP period. However, the proportion of intrauterine growth restriction (IUGR) (0.2% to 0.7%) and gestational diabetes mellitus (GDM) (1.7% to 15.6%) was significantly (*p* < 0.05) increased over the policy changes. After adjusting for confounding factors, only the risk of GDM increased [aOR 10.91 (95% CI: 6.05, 19.67)] in women with AMA from the OCP period to the universal TCP period. In conclusion, the risk of GDM increased in women with AMA from the OCP period to the universal TCP period.

## Introduction

The Chinese government has been implemented an OCP since 1979, to control the rising population growth rate (540 million to 800 million during 1950–1970) and reduce the fertility rate^1^. This policy encouraged delayed childbearing, longer interpregnancy intervals, and fewer children which have stopped around 4000 million babies, and as a result, the birth rate has dropped significantly between 1.5 and 1.7 births per woman and has remained the same till now^2^. Meanwhile, the OCP had a profound impact on China’s population, societal, and health consequences, such as imbalance of sex ratio at birth, aging of the population, reduction of the workforce, and rising rate of C-section deliveries^3–6^.

In 2013, the Chinese government declared a partial TCP, allowing married couples to have a second child if either parent was a singleton. By easing certain restrictions in the OCP, the National Health and Family Planning Commission declared that the partial TCP would increase the supply of the workforce, reduce the aging population and sex-ratio imbalance. Following this, the Chinese government officially announced a universal TCP in October 2015, the 36-year OCP came to end^2^. According to hospital surveillance data, the number of live births had increased to 17.86 million per year by 2016, reflecting a 7.9% increase over 2015^7^. After the universal TCP, the number of deliveries with obstetric complications and the number of pregnant women with elder maternal age increased^8^. However, long-term studies to investigate the maternal and neonatal health consequences especially in women with the AMA after the implementation of TCP are limited.

It has been estimated that after the universal TCP, an additional 90 million women in China will have a second child. Of these pregnant women, 60% are estimated to be older than 35 years, and 50% will be 40 years old^9^. It is well-established that women aged 35 years or older had a significantly increased risk of preeclampsia (PE), placenta previa, and adverse perinatal outcomes^10^. A retrospective study revealed that the trend of AMA increased in the Chinese population and was associated with higher odds of C-section, preterm births, low Apgar score, and perinatal mortality^11^. Moreover, as a result of universal TCP, the expected increasing number of older pregnant women will pose an extra challenge for health caregivers and obstetricians and can further increase maternal-neonatal consequences^9^.

The policy change has affected many aspects of China’s population and maternal-neonatal health. Several studies have investigated the impact of universal TCP on maternal-neonatal outcomes. For example, the impact of birth policy changes on pregnancy outcomes^8^, the sex ratio at birth^3^, maternity care^9^, maternal risk factors and pregnancy complications^12^, C-section rate^13^, women’s reproductive decision^14^, maternal-neonatal outcomes^15^, and prevalence of congenital defect^7^ has been reported. However, limited studies have reported the effect of universal TCP on maternal age pregnancy outcomes in women with AMA^7,12^. Therefore, we aimed to examine changes in maternal age and adverse pregnancy outcomes in women with AMA after the implementation of a universal TCP in Hubei province, China.

## Results

### Changes in maternal characteristics and complications

Among the total births (n = 23,051), 5653 births were in the OCP period (2011–2013), 8137 births in the partial TCP period (2014–2016), and 9261 births in the universal TCP period (2017–2019). The proportion of neonatal births to women aged < 30 decreased, whereas the proportion of neonatal births to women aged 30–34 years and women with AMA increased from OCP to universal TCP period. In total, 3956 (17.2%) births were to women with AMA. The proportion of neonatal births to women with AMA increased by 68.8%, from 12.5% in 2011–2013 to 21.1% in 2017–2019. The proportion of births to multiparous women increased from 18.5% in the OCP period to 27.7% in the universal TCP period. Pregnancy complications including HDP, abnormal placentation, and GDM significantly increased over the policy changes (p < 0.05). Moreover, after adjusting for confounding factors, the risk of AMA significantly increased in the universal TCP period relative to the OCP period (Tables 1, and 2).

**Table 1:** Changes in maternal characteristics from OCP (One-child policy) period to universal TCP (Two-child policy) period. * = Frequency and percentage of variables with only ‘Yes’ value presented**,** AMA (advanced maternal age) HDP (Hypertensive disorders of pregnancy composite of Gestational hypertension (GH), Preeclampsia (PE) and severe PE), Abnormal placentation (Composite of placenta previa and placental abruption), GDM (Gestational diabetes mellitus), *p-values* were calculated using chi-square test.

Maternal age Characteristics	OCP period (2011–2013) N %		Partial TCP period (2014–2016) N %		Universal TCP period (2017–2019) N %		Cramer's V	*P*-value
Total	5653	100.00	8137	100.00	9261	100.00	–-	–-
< 30	3232	57.1	4252	52.2	3836	41.4	0.09	< 0.001 (trend )
30–34	1716	30.4	2584	31.8	3475	37.5		
≥ 35 (AMA)	705	12.5	1301	16.0	1950	21.1		
**Maternal Education**								
Low	1432	25.3	1926	23.7	1584	17.2	0.07	< 0.001
Middle	2241	39.6	3226	39.6	3607	38.9		
Higher	1980	35.1	2985	36.7	4070	43.9		
**Maternal occupation**								
Housewives	3153	55.8	4328	53.2	4561	49.3	0.03	< 0.001
Professional services	2363	41.8	3624	44.5	4475	48.3		
Manual workers	137	2.4	185	2.3	225	2.4		
**Parity**								
Primiparous (≤ 1)	4606	81.5	6181	76.0	6696	72.3	0.08	< 0.001
Multiparous (> 1)	1047	18.5	1956	24.0	2565	27.7		
C-section*	3276	58.0	5049	62.0	5681	61.3	0.03	< 0.001
Previous history of C-section*	366	6.5	1234	15.2	1993	21.5	0.16	< 0.001
HDP*	313	5.5	458	5.6	725	7.8	0.04	< 0.001
Abnormal placentation*	187	3.3	348	4.3	480	5.2	0.03	< 0.001
GDM*	41	0.7	409	5.0	1088	11.7	0.17	< 0.001

**Table 2:** Association between maternal age and policy changes. OCP (One-child policy), TCP (Two-child policy), AMA (advanced maternal age), Adjusted for maternal education, occupation, and parity; * = *P* < *0.05.*

Maternal age (years)	OCP period (2011–2013) aOR (95% CI)	Partial TCP period (2014–2016) aOR (95% CI)	Universal TCP period (2017–2019) aOR (95% CI)
< 30	1.00 (reference)	0.86 (0.80, 0.92)*	0.57 (0.53, 0.61)*
30–34	1.00 (reference)	1.04 (0.96, 1.12)	1.31 (1.21, 1.41)*
≥ 35 (AMA)	1.00 (reference)	1.27 (1.15, 1.40)*	1.76 (1.60, 1.93)*

### Changes in neonatal outcomes

The proportion of neonates with preterm births, LBW, low Apgar scores, fetal distress, and congenital defects significantly decreased from 19.2%, 14.2%, 3.3%, 4.2%, and 1.4% in the OCP period to 18.3%, 13.2%, 3.0%, 2.4%, and 1.0% in the universal TCP period, respectively. However, perinatal mortality and neonates with IUGR significantly increased (*p* < 0.05) from the OCP period to the universal TCP period (Table 3).

**Table 3:** Changes in neonatal characteristics from OCP (One-child policy) period to universal TCP (Two-child policy) period. * = Frequency and percentage of only ‘Yes’ value presented**,** LBW (Low birth weight), IUGR (Intrauterine growth restriction), LPI (Low ponderal index), congenital defects (microtia, anotia, polydactyly, heart defects, limb reduction defects, cleft lip, cleft palate, hydrocephaly, and NTDs), *p-values* were calculated using chi-square test.

Neonatal characteristics	OCP period (2011–2013)		Partial TCP period (2014–2016)			Universal TCP period (2017–2019)		Cramer's V		*P*-value
	N	%	N	%		N	%			
Total	5653	100.00	8137	100.00		9261	100.00	–-		–-
Preterm births*	1084	19.2	1635	20.1		1693	18.3	0.02		0.01
Perinatal mortality*	81	1.4	43	0.5		204	2.2	0.06		< 0.001
LBW*	802	14.2	1248	15.3		1221	13.2	0.02		< 0.001
IUGR*	19	0.3	31	0.4		118	1.3	0.05		< 0.001
LPI*	216	3.8	325	4.0		354	3.8	0.01		0.8
Low Apgar score*	184	3.3	372	4.6		282	3.0	0.03		< 0.001
Fetal distress*	240	4.2	60	0.7		221	2.4	0.09		< 0.001
Fetal macrosomia*	306	5.2	466	5.7		480	5.2	0.01		0.2
Congenital defects*	79	1.4	124	1.5		95	1.0	0.02		0.01
**Neonatal gender**										
Male	3038	53.7	4385	53.9		4902	52.9	0.01		0.4
Female	2615	46.3	3752	46.1		4359	47.1			

### Changes in adverse pregnancy outcomes in women with AMA

Among adverse pregnancy outcomes in women with AMA, the proportion of preterm births (from 29.4% to 24.1%), LBW (from 20.9% to 15.9%), low Apgar score (from 4.8% to 3.6%), fetal distress (from 4.5% to 2.2%), HDP (from 11.5% to 9.2%), and C-section (from 73.5% to 71.8%) significantly (p < 0.05) decreased from the OCP period to the universal TCP period. However, the proportion of IUGR (from 0.2% to 0.7%) and GDM (from 1.7% to 15.6%) significantly (p < 0.05) increased over the policy changes. Compared to the OCP period, after adjusting for confounding factors, the risk of GDM increased [aOR 10.91 (95% CI: 6.05, 19.67)] in women with AMA in the universal TCP period (Tables 4 and 5).

**Table 4:** Changes of adverse pregnancy outcomes in women with AMA over the period of policy changes. OCP (One-child policy), TCP (Two-child policy), AMA (advanced maternal age), LBW (low birth weight), IUGR (intrauterine growth retardation), LPI (low ponderal index), HDP (hypertensive disorders of pregnancy), GDM (gestational diabetes mellitus).

Adverse pregnancy outcomes	OCP period (2011–2013) (N = 705) N %		Partial TCP period (2014–2016) (N = 1301) N %		Universal TCP period (2017–2019) (N = 1950) N %		Cramer's V	P-value
Preterm births	207	29.4	336	25.8	470	24.1	0.04	0.02
Perinatal mortality	21	3.0	11	0.8	58	3.0	0.06	< 0.001
LBW	147	20.9	240	18.4	310	15.9	0.04	0.008
IUGR	2	0.2	7	0.5	14	0.7	0.02	0.03
LPI	33	4.7	58	4.5	75	3.8	0.01	0.5
Low Apgar score	34	4.8	77	5.9	71	3.6	0.04	0.009
Fetal distress	32	4.5	14	1.1	43	2.2	0.08	< 0.001
Macrosomia	35	5.0	73	5.6	109	5.6	0.01	0.04
Congenital defects	15	2.1	16	1.2	23	1.2	0.03	0.15
HDP	81	11.5	100	7.7	180	9.2	0.04	0.01
Abnormal placentation	44	6.2	93	7.1	122	6.3	0.01	0.5
GDM	12	1.7	113	8.7	305	15.6	0.17	< 0.001
C-section	518	73.5	1004	77.2	1400	71.8	0.05	0.003

**Table 5:** Association between adverse pregnancy outcomes in women with AMA and policy changes. OCP (One-child policy), TCP (Two-child policy), AMA (advanced maternal age), LBW (low birth weight), IUGR (intrauterine growth retardation), LPI (low ponderal index), HDP (hypertensive disorders of pregnancy), GDM (gestational diabetes mellitus), Adjusted for maternal education, occupation, pre-pregnancy body weight, parity, and neonatal gender; * = *P* < *0.05.*

Adverse pregnancy outcomes	OCP period aOR (95% CI)	Partial TCP period aOR (95% CI)	Universal TCP period aOR (95% CI)
Preterm births	1.00 (reference)	0.89 (0.73, 1.10)	0.88 (0.72, 1.08)
Perinatal mortality	1.00 (reference)	0.31 (0.14, 0.64)	1.25 (0.73, 2.14)
LBW	1.00 (reference)	0.94 (0.75, 1.19)	0.88 (0.70, 1.11)
IUGR	1.00 (reference)	4.46 (0.55, 36.08)	5.99 (0.76, 47.08)
LPI	1.00 (reference)	0.95 (0.61, 1.49)	0.82 (0.53, 1.27)
Low Apgar score	1.00 (reference)	1.33 (0.87, 2.01)	0.87 (0.56, 1.34)
Fetal distress	1.00 (reference)	0.24 (0.12, 0.45)*	0.52 (0.32, 0.85)*
Macrosomia	1.00 (reference)	1.11 (0.73, 1.69)	1.14 (0.76, 1.71)
Congenital defects	1.00 (reference)	0.66 (0.32, 1.36)	0.70 (0.35, 1.39)
HDP	1.00 (reference)	0.69 (0.50, 0.94)*	0.85 (0.63, 1.14)
Abnormal placentation	1.00 (reference)	1.16 (0.80, 1.69)	1.06 (0.73, 1.53)
GDM	1.00 (reference)	5.53 (3.02, 10.01)*	10.91 (6.05, 19.67)*
C-section	1.00 (reference)	1.19 (0.96, 1.47)	0.83 (0.68, 1.01)

## Discussion

In the present tertiary hospital-based retrospective study (2011–2019), we found changes in maternal age and adverse pregnancy outcomes in women with AMA after the implementation of the universal TCP. We observed that the proportion of neonatal births to women with AMA increased from the OCP period to the universal TCP period. The risk of AMA significantly increased in the universal TCP period compared with the OCP period. In the univariate analysis, among adverse pregnancy outcomes in women with AMA, the proportion of preterm births, LBW, HDP, and C-sections significantly decreased from the OCP period to the universal TCP period. However, the proportion of IUGR, fetal macrosomia, and GDM significantly increased over the policy changes. After adjusting for confounding factors, relative to the OCP period, the risk of GDM increased in women with AMA in the universal TCP period.

Our finding indicated a sharp increase (68.8%) in the proportion of women with AMA over the birth policy changes (from 12.5% in the OCP period to 21.1% in the universal TCP period). Although delayed childbearing has increased across the globe ^16,17^, the universal TCP in China might have encouraged the desire for a second child among older women. Based on the expected estimate, the universal TCP will encourage an additional 90 million women to have a second child and among these women, 60% will be older than 35 years ^9^. In accordance with our findings, some studies in China reported the impact of universal TCP on maternal age. For example, Li et al. ^15^ found that after implementing the universal TCP, around 5.40 million neonates were born between July 2016 and December 2017. During the study period, 9.1% and 5.8% monthly mean increase was found in multiparous mothers and mothers with AMA, respectively.

In Zhejiang Province, ^7^ women with AMA increased from 8.52% in 2013 to 15.82% in 2017. A sharp increase was found in births to women with AMA from 2013 to 2017. The new China’s universal TCP may have increased the tendencies toward fertility desires among older women. These findings were also evidenced in the Chinese national surveillance data indicating that after the relaxation of OCP, women with AMA were increased from 7.8 to 10.9% ^18^.

In Beijing, after the enactment of universal TCP, women of older maternal age (≥ 40 years) increased significantly from 2.2 to 3.6%^8^. In Hebei province^3^, the proportion of pregnant women aged ≥ 30 years increased from 24.7% (in the OCP period) to 36.9% (in the universal TCP period). It indicates that the introduction of universal TCP encouraged women of old age to have a second child which was prohibited before the implementation of the birth policy change. We speculate that the increase in AMA could be in multiparous women. The proportion of multiparous women has increased from the OCP period to the universal TCP period, showing that women who previously had no plans to have an additional child during their childbearing age chose to have a second child ^8,15^.

Based on the evidence, the implementation of universal TCP has caused a significant increase in the proportion of elderly parturient women^3,7,8,15^. These women of old age are likely to be associated with an increased risk of pregnancy complications such as HDP, abnormal placentation, GDM, C-section, and adverse perinatal outcomes^19^. In the univariate analysis, we observed that in women with AMA, the proportion of preterm births, LBW, C-section, and HDP were significantly declined from the OCP period to the universal TCP period. Moreover, after adjusting for confounding factors, the risks of these adverse pregnancy outcomes in women with AMA were not significantly increased after the implementation of the universal TCP. Zhang et al.^8^ reported that HDP, placenta previa, placental implantation, and severe postpartum hemorrhage were significantly increased after the introduction of universal TCP compared with the OCP period. They believed that the increase in pregnancy complications could be due to AMA because a large number of older women became pregnant after the implementation of universal TCP and the proportion of women with AMA increased.

However, our univariate analysis showed that although the proportion of women aged ≥ 35 years increased but, the proportion of some adverse perinatal outcomes (i.e. preterm births, LBW), HDP, and C-sections were significantly decreased in women with AMA after the announcement of the universal TCP. The improvement of these pregnancy outcomes regardless of the impact of AMA could be attributed to improvement in socioeconomic status (SES), health sector initiatives, and investment in health care services. We observed that SES in women with AMA improved from the OCP period to the TCP period (table S1). Women of higher SES status were associated with a lower incidence of LBW in Shaanxi, China^20^. Similar associations between maternal SES and pregnancy outcomes were reported in many previous studies^21,22^.

The Chinese government has increased health expenditure per capita from US$ 53 in 1995 to US$ 480 in 2012 and achieved remarkable goals in the last two decades^23^. To better deliver and manage the basic public health services, the Chinese government issued three editions of National Basic Public Service Specifications in 2009, 2011, and 2017, respectively. These service packages of the program consist of health education, health management of children aged 0–6, and maternal health care^24^. Therefore, the strengthening and improvement of the three-tier medical and health services network for pregnant women in China has proven to decline the trends of adverse perinatal outcomes^25^.

In the univariate analysis we showed that after the implementation of universal TCP, the proportion of GDM significantly increased in women with AMA. Moreover, after adjusting for confounding factors the risk of GMD significantly increased by tenfold in women with AMA in the universal TCP period. It is obvious that an increase in maternal age results in a higher incidence of GDM. In our findings, the proportion of GDM in women with AMA increased from 1.7% (in the OCP period) to 15.6% (in the universal TCP period). Zhang et al.^8^ also found that GDM increased from 23.1% (in the OCP period) to 25.3% (in the universal TCP period). However, their study did not report the proportion of GDM in women with AMA. A retrospective study conducted by Teng et al.^12^ examined changes in maternal age and pregnancy outcomes after the universal TCP. They reported that the incidence of GDM increased from 18.72% in 2015 to 25.59% in 2017. The proportion of GDM in women with AMA increased from 3.90% in 2015 to 5.48% in 2016 and then decreased to 0.35% in 2017.

AMA is an independent risk factor for GDM^26^. As maternal age increases, the prevalence of GDM increases simultaneously. The prevalence of GDM in women aged < 25 years, 25–29 years, 30–34 years, 35–39 years and > 40 years, were 6.6%, 7.3%, 8.8%, 16.7%, and 35.2%, respectively ^27^. After adjusting for potential confounding factors, the risk of GDM increased with maternal age ^28^. In the Chinese population, AMA increased the risk of GMD by 4.8-fold^29^. Moreover, in another Chinese population, women with AMA associated with a 3.6-fold increased risk of GDM^30^. The higher risk of GDM in women with AMA could be explained by the progressive vascular endothelial damage in women of older ages^31^, reduction in insulin sensitivity, impaired glucose tolerance^32^, and deterioration of pancreatic β-cell function ^33^ as maternal age increases.

Moreover, in our findings, the higher risk of GDM in women with AMA could be due to a sedentary lifestyle and higher body mass index (BMI). In general, women of professional services have higher tendencies of a sedentary lifestyle. In our findings, in women with AMA, the trend of professional services (i.e. doctors, nurses, accountants, teachers, lawyers, and actresses) increased from 27.1% in the OCP period to 49.1% in the universal TCP period (table S1). A sedentary lifestyle has become a significant problem across the globe and has been linked with a range of chronic health condition^34^ including metabolic dysfunction and impaired blood sugar regulation^35^. In the Brazilian pregnant women population, the sedentary lifestyle was associated with higher odds of GMD after adjusting for several confounding factors (aOR 1.9-fold)^36^. Similarly, in a population-based cross-sectional study the sedentary lifestyle was associated with a higher risk of GDM among Chinese pregnant women^37^. The high BMI of pregnant women is associated with an increased risk of GMD^38^. However, our study lacks data on the BMI of pregnant women which is one of the limitations of our study.

Our study had certain limitations which are worth mentioning. The study design was retrospective. Our data analysis is based on a single-center tertiary hospital, which is the potential selection bias in this study. We also excluded the women with chronic hypertension which could limit the generalizability of our findings. Moreover, we could not distinguish the C-section whether it was an elective or an emergency C-section. As a tertiary-level hospital, many pregnant women with severe pregnancy complications are transferred to our hospital, resulting in a relatively high C-section rate in women with AMA. Therefore, our results cannot be generalized to the whole population and pregnant women living in other regions of China.

## Conclusion

In summary, the proportion of neonatal births to women with AMA increased from the OCP period to the universal TCP. The risk of AMA significantly increased in the universal TCP relative to the OCP period. After adjusting for confounding factors, the risk of GDM increased in women with AMA in the universal TCP period compared with the OCP period. As we were working on the impact of TCP on maternal age and adverse pregnancy outcomes in women with AMA, the Chinese government announced the tree-child policy^39^. Therefore, this study will help obstetricians and clinicians to pay serious attention to the increasing trend of AMA and associated adverse pregnancy outcomes after the three-child policy.

## Material and methods

### Study population

A tertiary hospital-based retrospective study was conducted in the Wuhan University Renmin Hospital, Department of Obstetrics and Gynecology, Hubei, China from January 2011 to December 2019. The data was collected and documented in the obstetrics register and electronic database by trained nurses during individual examinations in the Gynecology and Obstetrics Department. The study protocol was approved by the Ethical Review Board of Renmin Hospital (ID: WDRY2019–K034) in accordance with the Declaration of Helsinki. The need for informed consent, according to national legislation, was waived by the Ethical Review Board of Renmin Hospital because this was a retrospective cohort study.

### Inclusion and exclusion criteria

**Figure 1:**
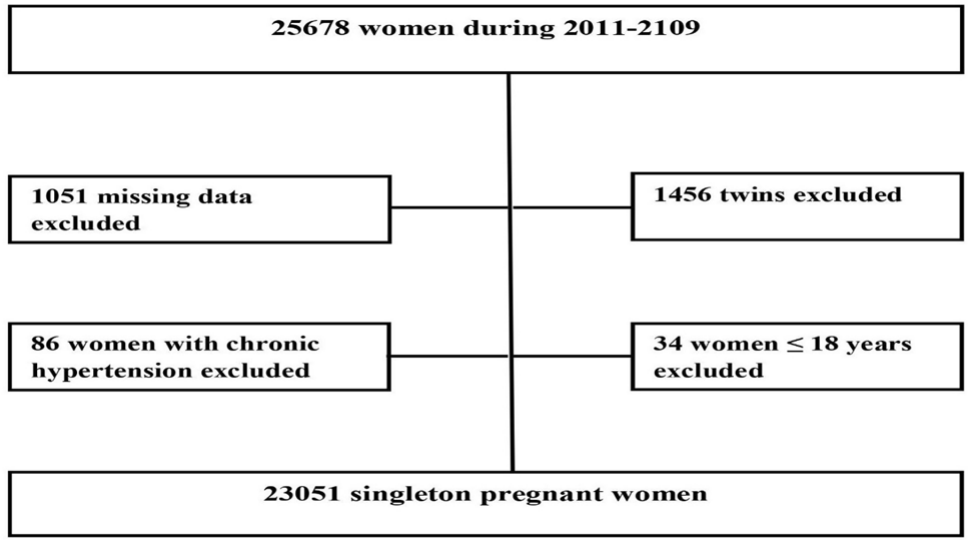
Flow chart of study population.

A total of 23,051 singleton pregnant women were selected for the study. We excluded missing data on maternal age, pre-pregnancy body weight, neonatal gender, birth weight, birth length, and gestational age^40^. Pregnant women aged ≤ 18 years old, with chronic hypertension, and twin neonates were also excluded from the data analysis as shown in Fig. 1.

### Collection of data on maternal traits

Data regarding maternal traits were collected from the obstetrics register including maternal age, parity, prepregnancy body weight, gestational age, education, occupation, and pregnancy complications. At the time of delivery, based on age, pregnant women were divided into three groups (i) < 30 years, (ii) 30–34 years, (iii), and ≥ 35 years. Gestational age was calculated by the date of the last known menstrual period and confirmed by ultrasound examination during the first and second trimesters. Based on education, they were classified as ≤ 8 years (low), 9–12 years (middle), and ≥ 13 years (higher). Maternal occupations were categorized as (i) housewives, (ii) professional services (doctors, nurses, accountants, teachers, lawyers, and actresses), and (iii) manual workers (farmers, waitresses, drivers, and factory workers).

### Definition of pregnancy complications and perinatal birth outcomes

Gestational hypertension (GH) is defined as having blood pressure greater than 140/90 mmHg without proteinuria after 20th weeks of gestation^41^. PE is defined as elevated blood pressure 140/90 mmHg with proteinuria (albumin > 0.3 g in 24 h) after the 20th week of gestation^42^. Sever PE referred to having a blood pressure higher than 160/110 mmHg with proteinuria (albumin > 5 g in 24 h) after the 20th week of gestation^43^. Placenta previa is defined as suboptimal placental implantation near or over the cervical opening^44^. Placental abruption referred to the early separation of the placenta before childbirth^45^. Neonatal birth outcomes were recorded immediately after neonatal birth including birth weight in grams using an electronic infant scale, birth length in centimeter using a standard measuring board for the neonate. Preterm birth is defined as a neonate born before 37 completed weeks or fewer than 259 days from the first date of a woman’s last menstrual period^46^. Perinatal mortality is defined as the combination of late fetal mortality (stillbirths) and early neonatal mortality (0–6 days of life)^47^. Fetal macrosomia is defined as birth weight ≥ 4000 g and LBW is defined as birth weight < 2500 g^48^. IUGR is defined as a condition of fetal growth that is below the 10th percentile for its gestational age and does not reach its genetically predetermined growth potential^49^. Apgar score was determined by evaluating the newborn baby on five simple criteria on a scale from zero to two, then summing up the five values obtained. Apgar score was recorded at 1 min and 5 min after birth. Apgar score was divided into two categories (i) low Apgar score (< 7), and (ii) normal Apgar score (≥ 7)^50^. Fetal distress is defined as a pathophysiological condition in which the fetus is suffering from insufficient oxygen supply^51^. The ponderal index was determined by weight in gm / (length in cm)^3^  × 100. The ponderal index between 2.5 and 3.0 was considered normal between 2.0 and 2.5 marginal, and a neonate with a ponderal index less than 2.0 was considered a low ponderal index (LPI)^52^. Congenital defects are defined as abnormalities in the structure of neonatal body parts that occur during intrauterine development^53^.

### Potential confounding factors

Cofounding factors were selected based on previous literature which is associated with both exposure and perinatal birth outcome y^42^. The confounding factors included in this analysis were maternal education, occupation, pre-pregnancy body weight (≤ 45 kg and ≥ 91 kg), parity, and neonatal gender.

### Statistical analysis

The categorical and binary variables are presented as number (n) and percentage (%). We divided the study period into OCP (2011–2013), partial TCP (2014–2016), and universal TCP (2017–2019). Changes in maternal age and maternal-neonatal factors in women of AMA were estimated using chi-square tests. To estimate the effect size, Cramer’s V was used in chi-square tests. Trend analysis (chi-square tests) was used to estimate the change in maternal age over the time period of policy changes. Additionally, we restricted our analysis to women aged ≥ 35 years to know changes in pregnancy complications and adverse perinatal outcomes in women with AMA over the time frame of policy changes. Binary logistic regression analysis was used to calculate odds ratios (ORs) and 95% confidence interval (CI) to examine the association of universal TCP with the risk of changes in maternal age groups and risk of pregnancy complication and adverse perinatal outcomes in women with AMA, while the OCP period being defined as the reference period. *P* < 0.05 was taken statistically significant. The data were analyzed using SPSS (Statistical Package for Social Sciences) for window version 22 (IBM Corporation, Chicago, USA).

### Ethics approval and consent to participate

The study protocol was approved by the Ethical Review Board of Renmin Hospital (ID: WDRY2019–K034) in accordance with the Declaration of Helsinki.

### Informed consent

The need for informed consent, according to national legislation, was waived by the Ethical Review Board of Renmin Hospital because this was a retrospective cohort study.

## Supplementary Information

Supplementary Information.

## Data Availability

All data analyzed during this study are included in this article.
