# Supplementary material for: Changes in adverse pregnancy outcomes in women with advanced maternal age (AMA) after the enactment of China’s universal two-child policy
Source: Sci Rep. 2022 Mar 23;12:5048. doi: 10.1038/s41598-022-08396-6 (PMC8943149; doi:10.1038/s41598-022-08396-6)
Supplement: Supplementary file 1 — Supplementary Information. [file 41598_2022_8396_MOESM1_ESM.docx]

| Socioeconomic status | OCP period  (2011-2013) (N= 705)  N % | | Partial TCP period  (2014-2016) (N= 1301)  N % | | Universal TCP period  (2017-2019) (N= 1950)  N % | | Cramer's V | P-value |
| --- | --- | --- | --- | --- | --- | --- | --- | --- |
| Maternal Education |  |  |  |  |  |  |  |  |
| Low | 288 | 40.9 | 354 | 27.2 | 330 | 16.9 | 0.05 | <0.001 |
| Middle | 268 | 38.0 | 484 | 37.2 | 772 | 39.6 |  |  |
| Higher | 149 | 21.1 | 463 | 35.6 | 848 | 43.5 |  |  |
| Maternal occupation |  |  |  |  |  |  |  |  |
| Housewives | 495 | 70.2 | 709 | 54.5 | 942 | 48.3 | 0.06 | <0.001 |
| Professional services | 191 | 27.1 | 561 | 43.1 | 958 | 49.1 |  |  |
| Manual workers | 19 | 2.7 | 31 | 2.4 | 50 | 2.6 |  |  |

**Table S1.** Changes of socioeconomic status in women with AMA over the period of policy changes. OCP (One-child policy), TCP (Two-child policy),
